# Antimicrobial activity of synthetic cationic peptides and lipopeptides derived from human lactoferricin against *Pseudomonas aeruginosa* planktonic cultures and biofilms

**DOI:** 10.1186/s12866-015-0473-x

**Published:** 2015-07-07

**Authors:** Susana Sánchez-Gómez, Raquel Ferrer-Espada, Philip S. Stewart, Betsey Pitts, Karl Lohner, Guillermo Martínez de Tejada

**Affiliations:** Department of Microbiology, University of Navarra, Irunlarrea 1, E-31008 Pamplona, Spain; Center for Biofilm Engineering, Montana State University, Bozeman, MT USA; Institute of Molecular Biosciences, Biophysics Division, University of Graz, Graz, Austria; Present address: Susana Sánchez-Gómez, Bionanoplus, 31110 Noain, Spain

**Keywords:** Antimicrobial peptides, Lactoferricin, *Pseudomonas aeruginosa*, Biofilm

## Abstract

**Background:**

Infections by *Pseudomonas aeruginosa* constitute a serious health threat because this pathogen –particularly when it forms biofilms – can acquire resistance to the majority of conventional antibiotics. This study evaluated the antimicrobial activity of synthetic peptides based on LF11, an 11-mer peptide derived from human lactoferricin against *P. aeruginosa* planktonic and biofilm-forming cells. We included in this analysis selected N-acylated derivatives of the peptides to analyze the effect of acylation in antimicrobial activity. To assess the efficacy of compounds against planktonic bacteria, microdilution assays to determine the minimal inhibitory concentration (MIC), minimum bactericidal concentration (MBC) and time-kill studies were conducted. The anti-biofilm activity of the agents was assessed on biofilms grown under static (on microplates) and dynamic (in a CDC-reactor) flow regimes.

**Results:**

The antimicrobial activity of lipopeptides differed from that of non-acylated peptides in their killing mechanisms on planktonic and biofilm-forming cells. Thus, acylation enhanced the bactericidal activity of the parental peptides and resulted in lipopeptides that were uniformly bactericidal at their MIC. In contrast, acylation of the most potent anti-biofilm peptides resulted in compounds with lower anti-biofilm activity. Both peptides and lipopeptides displayed very rapid killing kinetics and all of them required less than 21 min to reduce 1,000 times the viability of planktonic cells when tested at 2 times their MBC. The peptides, LF11-215 (FWRIRIRR) and LF11-227 (FWRRFWRR), displayed the most potent anti-biofilm activity causing a 10,000 fold reduction in cell viability after 1 h of treatment at 10 times their MIC. At that concentration, these two compounds exhibited low citotoxicity on human cells. In addition to its bactericidal activity, LF11-227 removed more that 50 % of the biofilm mass in independent assays. Peptide LF11-215 and two of the shortest and least hydrophobic lipopeptides, DI-MB-LF11-322 (2,2-dimethylbutanoyl**-**PFWRIRIRR) and DI-MB-LF11-215, penetrated deep into the biofilm structure and homogenously killed biofilm-forming bacteria.

**Conclusion:**

We identified peptides derived from human lactoferricin with potent antimicrobial activity against *P. aeruginosa* growing either in planktonic or in biofilm mode. Although further structure-activity relationship analyses are necessary to optimize the anti-biofilm activity of these compounds, the results indicate that lactoferricin derived peptides are promising anti-biofilm agents.

## Background

*Pseudomonas aeruginosa* is intrinsically resistant to many antibiotics, such as penicillins, first, second and third generation cephalosporines (except ceftazidime), tetracyclins and rifampicin. Resistance is due to the low permeability of its membrane, the presence of efflux pumps and the production of AmpC, a chromosomal β-lactamase [1]. Furthermore, this pathogen has a notorious ability to acquire additional mechanisms of resistance including those based on efflux pump overexpression, porin loss, alteration of drug target or enzymatic modification of antibiotics. This phenomenon frequently gives rise to clinical isolates displaying a multidrug-resistant phenotype that delays the appropriate antibiotic treatment and leads to therapeutic failure [1, 2].

The ability of *P. aeruginosa* cells to form biofilms during infection greatly facilitates its persistence inside the host and contributes to antibiotic resistance [3]. Biofilm formation first requires bacterial cell attachment to a surface followed by the development of a sessile colony with an extracellular matrix containing exopolysaccharide, proteins and nucleic acids. *P. aeruginosa* can form biofilms both in biotic (e.g. lung tissue in cystic fibrosis patients) and abiotic surfaces (e.g. indwelling medical devices). It is estimated that up to 65 % of bacterial infections are associated with the presence of biofilms [1–3]. In addition, biofilms are extremely resistant to antibiotics and immune system effectors [4, 5]. All these facts, underscore the importance of developing new therapies against biofilms formed by *P. aeruginosa*. Ideally, new drugs should have multiple mechanisms of action and low susceptibility to the development of resistance, compared to conventional antibiotics.

The antibiotic resistance of biofilm-producing bacteria has been attributed to a diversity of factors including the permeability barrier conferred by the exopolysaccharide, the altered chemical microenvironment within the biofilm (i.e. low oxygen concentration, waste product accumulation and acidic pH), the physiological heterogeneity of the bacterial population and the emergence of “persistent” cells [6].

Antimicrobial peptides (AMPs; e.g. nisin, indolicidin, cecropin, magainin, lactoferricin) are produced by a wide variety of organisms as a first line of defense. Their principal mechanism of action involves binding to conserved structural components of the bacterial envelope (e.g. lipopolysaccharide and lipoteichoic acid of Gram-negative and Gram-positive bacteria, respectively) followed by an interaction with the bacterial membrane that can be rapidly lethal. Some AMPs also bind to intracellular targets and inhibit essential biological processes including cell wall formation or DNA, RNA and protein synthesis [7, 8]. This mechanism of action is rapidly bactericidal and decreases the chances of resistance development compared to conventional antibiotics [9, 10].

The heterogeneity of bacteria embedded in the biofilm, a mixture of metabolically active and inactive cells, greatly reduces the efficiency of beta-lactams, which only kill actively dividing cells. In contrast, AMPs are bactericidal independently of the growing state of the target cell, (for a review see [11]) and this increases their appeal as potential anti-biofilm agents.

Lactoferricin is an AMP derived by pepsin digestion of lactoferrin, a multifunctional component of the innate immune system present in milk and other body fluids. Lactoferricin displays antimicrobial activity against a wide variety of microorganisms [12, 13]. Using rational design, we devised a peptide library based on the sequence of LF11, an 11-mer peptide derived from human lactoferricin, and determined the structural features governing the antimicrobial activity of the peptides against planktonic bacteria [14–17]. In the present article, we test the efficacy of these compounds against biofilms formed by *P. aeruginosa* under static and dynamic growth regimes. In these assays, we include acyl-derivatives of the parental compounds to deduce the contribution of the acyl group to the anti-biofilm activity.

## Results

### Antimicrobial activity on planktonic *P. aeruginosa* cells

To assess the antimicrobial activity of the peptides and lipopeptides against planktonic *P. aeruginosa* susceptibility assays consisting of MIC/MBC determination combined with killing kinetics studies were performed. Compounds displayed a wide range of antimicrobial efficacy (Table 1) with MICs varying from 8 to 128 μg/ml. Interestingly, except for two peptides, LF11-215 and LF11-322, the rest of the compounds were bactericidal at their MIC (i.e. MIC = MBC). As judged by MIC value, the least potent antimicrobials were the two acylated derivatives of peptide LF11-227, whereas a non-acylated compound, LF11-324, showed the best MIC.

**Table 1:** Relevant characteristics of peptides and lipopeptides used in this study

Peptides^a^	Sequences		MIC^b^ (μg/ml)	MBC^c^ (μg/ml)	T_3log_^d^ at different concentrations higher their MICs (minutes)			Hydrophobicity^e^ Δg_woct_ (kcal/mol)
					1X	2X	4X	
LF11-215		FWRIRIRR	64	128	>360	28	10	5,5
O-LF11-215	**octanoyl**	FWRIRIRR	32	32	17	21	10	3,2
DI-MB-LF11-215	**2,2-dimethylbutanoyl**	FWRIRIRR	16	16	17	11	10	3,2
6-MO-LF11-215	**6-methyloctanoyl**	FWRIRIRR	64	64	34	11	10	3,2
LF11-322		**P**FWRIRIRR	32	64	>360	11	11	5,64
DI-MB-LF11-322	**2,2-dimethylbutanoyl**	**P**FWRIRIRR	32	32	12	11	10	3,34
6-MO-LF11-322	**6-methyloctanoyl**	**P**FWRIRIRR	64	64	41	39	19	3,34
LF11-324		**PF**FWRIRIRR	8	8	113	13	10	3,93
LF11-227		FWRRFWRR	64	64	13	12	10	3,94
O-LF11-227	**octanoyl**	FWRRFWRR	128	128	10	10	10	1,64
6-MO-LF11-227	**6-methyloctanoyl**	FWRRFWRR	128	128	10	10	10	1,64

^a^: Peptide derivatives from human lactoferricin (based on residues 21–31), C-termini is amidated in all peptides; ^b^: Minimal inhibitory concentration against PAO1 planktonic cells; ^c^: Minimal bactericidal concentration against PAO1 planktonic cells; ^d^: T_3log_ is defined as the time needed to decrease 3 logs the initial inoculum determined in the killing curves assays. Relevant peptide modifications are shown in bold. ^e^:Peptide hydrophobicity is expressed as transfer free energy of peptides from water to n-octanol (ΔG_woct_) using Wimley-White octanol whole-residue scales [59] taking into account end group contributions, i.e. amidation of the C-termini and where appropriate acylation of N-termini. Latter was approximated by an acetyl group. Calculations were performed using MPEx [60]. Note that this parameter is inversely proportional to hydrophobicity

**Fig. 1:**
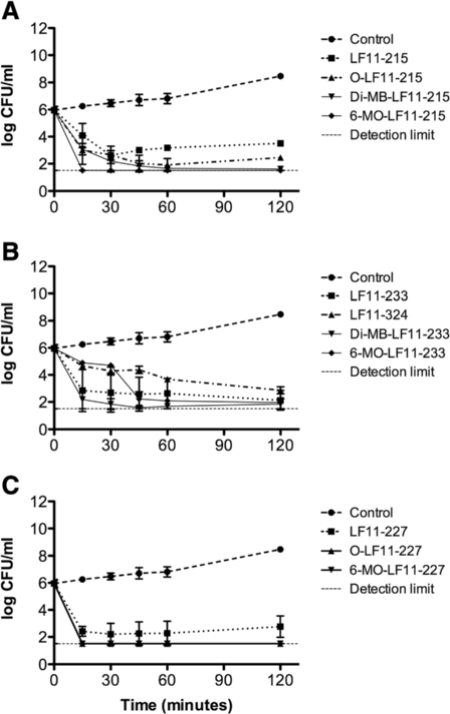
Time-killing curves of peptides and lipopeptides at their MBC against *P. aeruginosa* PAO1 strain. **a** LF11-215 and their N-acylated derivatives; (**b**) LF11-322 derivatives (amino acids insertion and acyl chain addition at N-termini); (**c**) LF11-227 and their N-acylated derivatives

In order to evaluate the bactericidal activity of the compounds, the kinetics of killing by each agent was measured at the following concentrations: 1, 2 and 4 times its respective planktonic MIC (1X, 2X and 4XMIC). Table 1 shows the time required by each compound to decrease 3 logs the initial inoculum (T_3log_). These assays revealed the rapid kinetics of killing mediated by most of the compounds. Notably, all peptides and lipopeptides except LF11-324 required less than 40 min at their MBC to reduce 3 log the initial inoculum. At 2 or 4×MIC, most of the agents exhibited a very rapid killing, with T_3log_ lower than 20 min. In accordance with these results, time-kill assays (Fig. 1) demonstrated that acylation resulted in an enhanced bactericidal activity, since compounds killed significantly faster than their parental non-acylated counterparts when all were tested at their respective MBC.

Compounds with the lowest MBC (e.g. LF11-324) did not correspond to those with the fastest killing kinetics. Paradoxically, agents that killed most rapidly were those lipopeptides that had been found to have the poorest MICs (O-LF11-227, 6-MO-LF11-227).

### Antimicrobial activity against *P. aeruginosa* biofilms

**Fig. 2:**
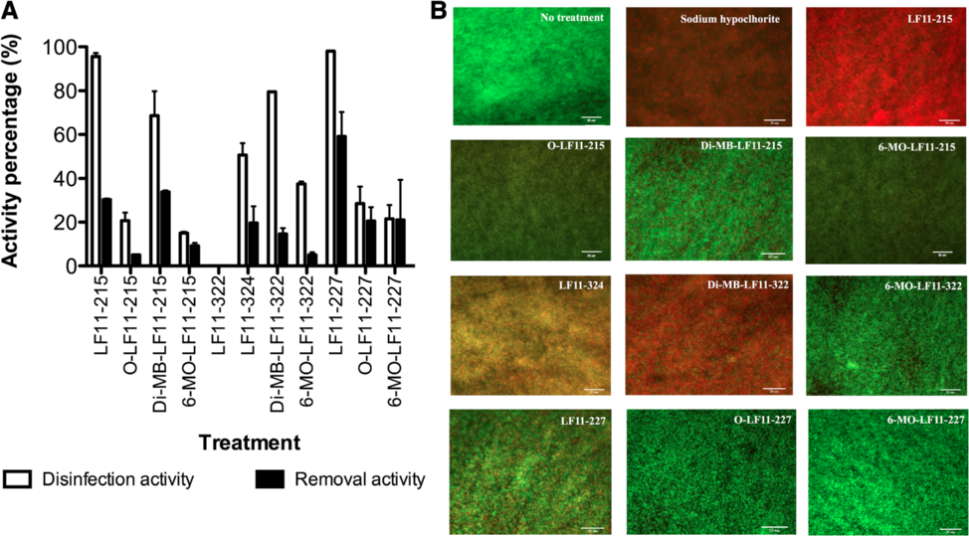
Effect of peptide treatment (at 10 times the planktonic MIC) on *P. aeruginosa* biofilms. Biofilms were grown in (**a**) microtiter plates (static conditions) and antibiofilm activities were determined by the MTT assay (disinfecting activity) and CV stain (removal activity); (**b**) CDC-reactor (turbulent conditions) and treated with 10 times the MIC of the indicated compound for 24 h at 37 °C. Live cells show green fluorescence due to GFP expression, whereas dead cells appear red because of the uptake of propidium iodide. Sodium hypochlorite at 500 μg/ml was used as an antimicrobial positive control. Scale Bars = 20 μm. LF-322 treated biofilms were not selected for fluorescence analysis since no activity against biofilm in microplates assays was found

The anti-biofilm activity of the compounds was determined on biofilms grown under static or dynamic flow regimes. On biofilms grown in microplates (i.e. static conditions), the disinfection and removal activities of the agents at 10XMIC were determined by assays based on respiratory rate (MTT test; 3-(4,5-dimethylthiazol-2-yl)-2,5-diphenyltetrazolium bromide), and biomass quantification (CV test; crystal violet test), respectively. Results of these experiments are summarized in Fig. 2a. Only LF11-227 and DI-MB-LF11-215 showed a removal activity higher than 35 %. Interestingly, two non-acylated compounds, LF11-215 and LF11-227, were found to be the most potent bactericidal agents killing almost 100 % of the biofilm forming bacteria after 24 h of incubation. Other peptides and lipopeptides (LF11-324, DI-MB-LF11-215 and DI-MB-LF11-322) displayed a medium-range disinfection activity, whereas LF11-322 showed no anti-biofilm activity.

The antimicrobial activity of the compounds was also evaluated on *P. aeruginosa* biofilms grown under dynamic flow conditions by fluorescent microscopy. For this purpose, biofilms were grown on coupons in a CDC-reactor until reaching a bacterial density of 7.63 log CFU/cm^2^ and then coupons were incubated during 60 min with 10XMIC of the corresponding compound. Viable cells of the strain used for these assays emit green fluorescence due to the expression of GFP (green fluorescence protein). To better distinguish between dead and live cells, PI (propidium iodide) was added to the samples. This dye only penetrates inside biofilms when cells are dead or damaged. When this occurs, PI interacts with DNA, and cells emit red fluorescence. Representative fluorescent micrographs of peptide and lipopeptide treated biofilms are shown in Fig. 2b. Despite the disparate methodologies and incubation times used for static and dynamically grown biofilm analysis (24 h vs. 60 min, respectively), several compounds were identified as having potent anti-biofilm activity under both conditions including LF11-215, LF11-324, DI-MB-LF11-322, and LF11-227. Due to the significant removal activity displayed by LF11-227 and DI-MB-LF11-215 (see above), it is possible that the microscopic analysis had underestimated the bactericidal activity of these two agents. The only acylated compounds that rivaled the anti-biofilm activity of. LF11-215 and LF11-227 were DI-MB-LF11-322 and DI-MB-LF11-215. Interestingly, those two lipopeptides share the same acyl group (2,2-dimethylbutanoyl). In striking contrast, the parental peptide LF11-322 displayed no anti-biofilm activity compared to their acylated derivatives.

After this preliminary screening, compounds with medium or high-range antimicrobial activity were selected to further characterize their activity against biofilms grown under dynamic conditions in the CDC-reactor. In these assays, mature biofilms were exposed to antimicrobials at different concentrations (1X, 2X and 10XMIC during 60 min or 10XMIC during 10 min) and the bactericidal effect was quantified by viable cell count and by confocal laser scanning microscopy (CLSM).

**Fig. 3:**
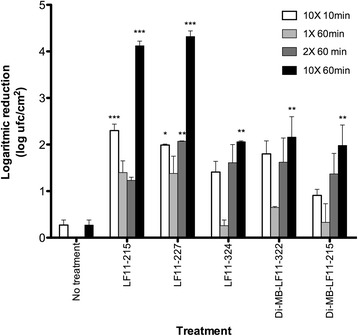
Log reduction of cell viability in *P. aeruginosa* biofilm grown in CDC-reactor. Concentrations used were 1, 2 and 10 times the planktonic MIC and biofilms were treated during 10 and 60 min. All experiments were performed in duplicate. ANOVA-Scheffé test was used to evaluate the statistical differences between the biofilm treated with the peptides and the untreated control incubated only with diluent buffer. *: *p* < 0.05; **: *p* < 0.01; and ***: *p* < 0.001

Viable count analysis, summarized in Fig. 3, showed that the anti-biofilm activity of compounds increases in a concentration-dependent manner. Overall, when tested on biofilms grown under dynamic conditions the antibacterial activity of the compounds was similar to that measured on statically cultured biofilms. LF11-215 and LF11-227 were the most potent peptides, since, when added at 10XMIC, they were able to decrease more than 2 logs (i.e. > 99 %) the cell viability of biofilms in just 10 min. Furthermore, both peptides caused a 10,000 fold reduction in biofilm cell viability after 1 h of treatment at 10XMIC.

Cytotoxicity of the compounds that displayed the best anti-biofilm activity was evaluated. LF-227, LF11-324, LF11-215 and its acyl derivative Di-MB-LF11-215, showed LC50 values higher than the maximum concentration assayed (10 times their MIC). On the contrary the lipopeptide Di-MB-LF11-322 was more cytotoxic (LC50 = 80 μg/ml, nearly 2XMIC value).

Compared to LF11-215 and LF11-227, the rest of the agents reduced viability to a lower extent and displayed a similar anti-biofilm activity regardless of the incubation time. The latter observation may reflect that these agents do not penetrate well inside the biofilm or that they display a rather slow killing mechanism on these structures.

**Fig. 4:**
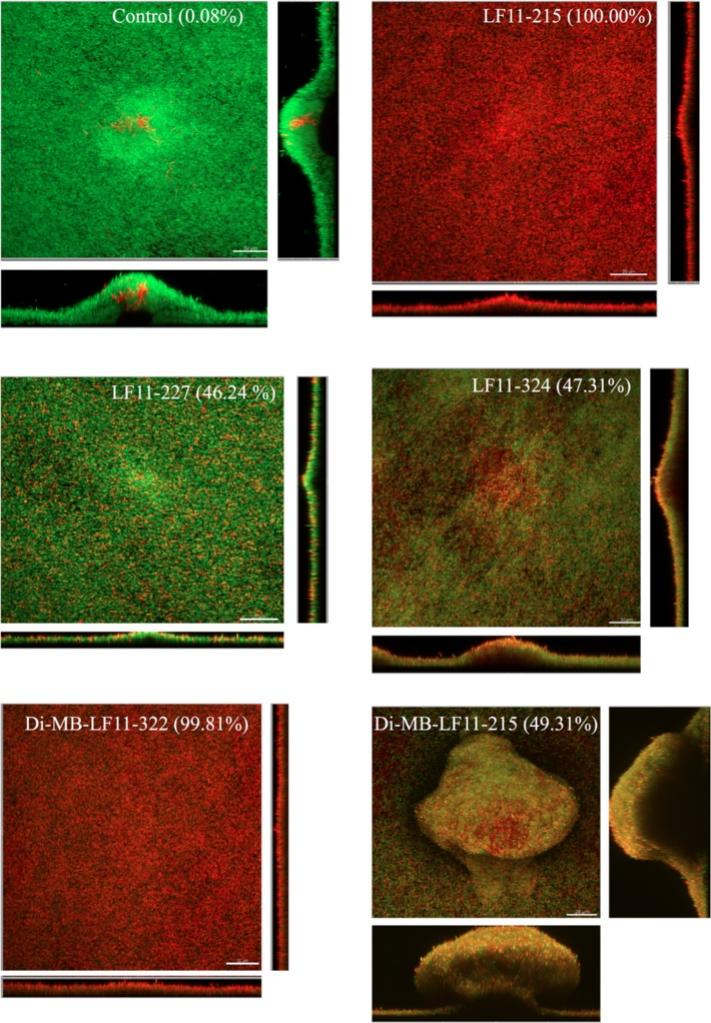
Bactericidal effect of peptides against *P. aeruginosa* biofilm grown in turbulent flow conditions (CDC-reactor). Antimicrobial activity was observed with CLSM. Live cells emit green fluorescence (due to GFP) and dead cells appear red (due to PI uptake). These images show control biofilm (no peptide treatment) and treated biofilm for 1 h at 10 times the MIC of peptides. The percentage shown in each image represented the percentage of dead cells (determined by volume). Scale Bars = 20 μm

To study these hypotheses, we treated biofilms with the selected compounds at 10XMIC for 60 min and then quantified by CLSM the percentage of viable (green) vs. dead (red) cells in the entire biofilm volume. As shown in Fig. 4, treatment with LF11-215 resulted in a uniform loss of viability in the whole biofilm structure (inner and outer layers; 100 % of red cells). Similar results were found with DI-MB-LF11-322 (99.81 %), although an inner layer of viable cells was detected. Compared to these two compounds, the rest of the peptides and lipopeptides displayed a lower anti-biofilm activity of similar magnitude (around 50 % of mortality). Finally, biofilms treated with LF11-324 and DI-MB-LF11-215 presented yellow cells. This phenomenon could be potentially explained by the existence of a cluster of permeabilized cells with reduced ability to exclude PI.

## Discussion

In the present study, we have identified short cationic peptides and lipopeptides derived from human lactoferricin that display rapid antimicrobial activity against *P. aeruginosa* planktonic and biofilm-forming cells. Some compounds penetrated deep into biofilm structures and killed homogenously even the inner layers of biofilm cells, as revealed by confocal microscopy analysis.

Acylation enhanced the bactericidal activity of the parental peptides and resulted in lipopeptides that were uniformly bactericidal at their MIC. The compound with the best inhibitory activity was peptide LF11-324 (MIC = 8 μg/ml). Interestingly, this antimicrobial activity did not vary when the compound was tested on the multiresistant *P. aeruginosa* clinical isolate PS4 [15]. Our results show that the presence of proline (P^1^) at the N-terminus of LF11-322 slightly improves peptide activity (in comparison with LF11-215). Similarly, the addition of another phenylalanine (F^2^) to LF11-322, greatly improves antimicrobial activity (see compound LF11-324) with an 8-fold increase in MIC (LF11-215 vs. LF11-324). These observations correlate with those of Zorko and collaborators, who analyzed the structure of peptide LF11-322 in lipidic environment [18]. According to these authors, the N-terminal residues P, F and W form an essential part of the peptide hydrophobic cluster, a region that upon insertion into the bacterial membrane determines the peptide ability to perturb the phospholipid bilayer.

The incorporation of an acyl group into the peptide structure resulted in a compound with improved inhibitory activity (MIC) only in the case of DI-MB-LF11-215, whereas the same acylation had a neutral impact on LF11-322 and LF11-227 activity. Although some authors reported that the N-acylation of LF11 with a lauryl group results in a compound with increased antimicrobial activity [19–21], we cannot extend this conclusion to the acylated derivatives tested here in terms of inhibitory activity.. Moreover, N-acylation enhances the binding to lipopolysaccharides of the outer membrane of Gram-negative bacteria to different degrees, which can counteract membrane permeabilization by reducing the effective peptide concentration at the inner membrane ([16, 22]). Another explanation for this discrepancy could be the fact that we used a Gram-negative organism in our assays. Thus, it has been demonstrated ([23]) that although peptide acylation could not improve the antimicrobial efficacy on Gram-negative organisms, it normally enhances this activity against Gram-positive bacteria. In agreement with this observation, we demonstrated that 6-MO-LF11-227 displayed a MIC eight times lower against *S. aureus* compared with its non-acylated counterpart, whereas such acylation did not improve antimicrobial activity against *P. aeruginosa* (data not shown).

It is likely that other features related with the spatial location or conformation of the substituent, the amphipathicity of the resulting lipopeptide or its interfacial activity have a great impact on the antimicrobial activity, as shown by others [24–27]. In addition, several authors reported that there is an optimum hydrophobicity window resulting in high antimicrobial activity and that any further increment or decline beyond that window dramatically decreases antimicrobial activity [28–30]. Similar observations were reported regarding the number of positive charges [24, 31].

Our results indicate that not only the acyl chain of lipopeptides plays an important role in the antimicrobial activity, but also the amino acid sequence. Time-kill assays performed with the 3 lipopeptides sharing identical acyl group (6-methyloctanoyl) revealed major differences in kinetics with compound 6-MO-LF11-227 being the fastest acting of all (Table 1 and Fig. 1). This agent has additional hydrophobic residues in its sequence (F and W) supporting the importance of hydrophobicity balance in antimicrobial activity, as discussed above.

Several studies reported the efficacy of AMPs or peptidomimetics against fungal and bacterial biofilms including those formed by *P. aeruginosa* [32–36]. Although lactoferrin is able to inhibit biofilm formation [37–39] and several investigations evaluated the inhibitory effects of derivatives of lactoferrin against biofilm generated by Gram-negative or Gram-positive organisms [40–45], to the best of our knowledge there are no reports characterizing the antimicrobial activity of lactoferrin derived peptides against *P. aeruginosa* biofilms.

To be useful against mature biofilms, antimicrobials must possess either disinfection activity (i.e. be bactericidal) or removal capacity. In assays on biofilms formed under static conditions (in microplates), we identified several peptides and lipopeptides that decreased more than 50 % cell viability in comparison with non-treated biofilms, whereas one peptide, LF11-227, was able to remove almost 60 % of the biofilm mass. It is possible that the high removal efficacy of this peptide (detected in microplate assays) may have prevented the visualization of more dead cells in fluorescence micrographs of LF11-227 treated biofilms. This phenomenon could also explain why DI-MB-LF11-215, another compound with a significant anti-biofilm activity, had an apparently low anti-biofilm activity when assessed by microscopy. Nevertheless, it is likely that the different methodologies used in those two types of assays, namely microplate (static conditions; 24 h of exposure) vs. CDC reactor (dynamic flow regime; 1 h) could explain the observed discrepancies in anti-biofilm activity.

Assays performed with biofilm grown under dynamic conditions confirmed that LF11-215 and LF11-227 were the agents with the most potent bactericidal activity being able to reduce 10,000 times the number of viable biofilm cells and displayed low cytotoxicity when tested at 10XMIC. Although those peptides have poor antimicrobial activity against planktonic cells (MIC = 64 μg/mL), they exert very potent anti-biofilm activity at only 10 times their planktonic MIC. Apart from direct killing, these agents might operate by additional uncharacterized anti-biofilm mechanisms. Thus, Fuentes-Nuñez and col. identified peptides that despite having high MICs displayed good anti-biofilm activity. These compounds acted by inhibiting a cellular stress response or by dysregulating genes related with biofilm formation, supporting the notion that antimicrobial and antibiofilm activity should be separately evaluated [46, 47].

We also observed regardless of the biofilm model used, that acylation led to a partial loss of the peptide anti-biofilm activity in the case of LF11-215 and LF11-227 derivatives. Interestingly, the lipopeptides that displayed the best activity against biofilms were DI-MB-LF11-322 and DI-MB-LF11-215, that share the same acyl group (2,2-dimethylbutanoyl), which were the smallest and among the least hydrophobic of the acylated compounds (see Table 1). Compared to other lipopeptides, it is likely that 2,2-dimethylbutanoyl bearing compounds could penetrate deeper into the biofilm matrix than the others. The low anti-biofilm efficacy of hydrophobic compounds could be due to their tendency to interact with EPS components of *P. aeruginosa* biofilms. Thus, high local concentrations of lipopeptides on EPS matrix may promote their aggregation and hinder their penetration. This limitation could theoretically explain the similar anti-biofilm activity detected with the least active peptides (DI-MB-LF11-322 and DI-MB-LF11-215 and LF11-324) at 2 and 10 times their MIC (Fig. 3). Among these peptides, Di-MB-LF11-322 showed certain toxicity, but other anti-biofilm treatment such as antibiotic lock therapy could be an alternative, since antibiotics are not in contact with tissues or patients bloodstream.

Moreover, LF11-324 treated biofilm showed the presence of yellow cells. This might indicate that although cell membranes are damaged, thus allowing the entry of PI, cells remain still alive and emit green fluorescence, explaining the yellow appearance. This permeabilizing effect was previously described by other authors using an unrelated peptide [48], that was reported to be partially bactericidal. This fact together with previous studies by our group that demonstrated synergistic combination between LF11-215 or LF11-227 and antibiotics in planktonic cultures [15], suggests that combination of lactoferricin derived peptides and antibiotics could enhance the efficacy of peptides as anti-biofilm agents [49]. The use of combinations of antimicrobials (AMPs and non-AMP related antimicrobials) as anti-biofilm therapy has already been successfully tested. This approach entailed not only the use of antimicrobials that target biofilm cells in different metabolic states [50, 51] but also the induction of antibiotic uptake due to the membrane disruption caused by another compound [52, 53].

## Conclusions

We identified peptides derived from human lactoferricin with potent antimicrobial activity against *P. aeruginosa* growing either in planktonic or in biofilm mode. In general, acylation of peptides increased the bactericidal activity against planktonic bacteria but reduced the anti-biofilm potency. Peptides and some of the least hydrophobic lipopeptides were able to kill efficiently biofilm forming cells of *P. aeruginosa* and to penetrate deep into the innermost layers of the biofilm matrix. Both peptides and lipopeptides displayed very rapid killing kinetics and all of them required less than 21 min to reduce 1,000 times the viability of planktonic cells when tested at 2 times their MBC.

Small and amphipathic cationic peptides are promising anti-biofilm agents, not only due to their broad activity spectrum, rapid mechanism of action and less susceptibility to resistance development, but also to their potential bactericidal activity against slow growing or even non-growing bacteria. Further structure-activity relationship analyses are necessary to optimize the anti-biofilm activity of lipopeptides.

## Methods

### Bacterial strains

*Pseudomonas aeruginosa* PAO1 was grown at 37 °C in LB (Luria Bertani, Pronadisa, Madrid, Spain) or TSB (tryptic soy broth, BioMériux, Marcy l’Etoile, France) broth or supplemented with agar (Pronadisa, Alconbendas-Madrid, Spain). When PAO1 pMF230 (GFP-expressing PAO1) [54] was grown, TSB was supplemented with 150 μg/ml of carbenicillin.

### Peptides

Peptides were synthesized with an amidated C-terminus by Polypeptide (Strasbourg, France) using 9-fluorenylmethyloxycarbonyl (Fmoc) solid phase chemistry and purified by RP-HPLC (Vydac C18 column; 0.1 % TFA/water; Beckman Coulter, Danvers, MA, USA). Mass spectroscopy analysis (Reflex IV, Bruker, Bremen, Germany) verified that their purity was >96 %. Peptides sequences are shown in Table 1.

### Susceptibility assays on planktonic cells

#### MIC and MBC determination

Minimal inhibitory concentrations (MIC) of the peptides were determined in Mueller Hinton (MH) medium (Difco Laboratories, Detroit, MI, USA) by the broth microdilution assay following recommendations of the Clinical and Laboratory Standards Institute (CLSI, formerly NCCLS) with some modifications [14]. Minimum bactericidal concentration (MBC) was defined as the concentration that caused a 3-log viability decrease in the initial inoculum and was calculated by colony counts on TSA plates incubated for 24 h at 37 °C.

#### Time-kill curves

Time-kill studies were determined with glass tubes containing 10 ml of MH and different concentrations of the peptides (1, 2 and 4 times their MIC). Overnight cultures of *P. aeruginosa* PAO1 in TSB were adjusted at 5 × 10^7^ CFU/ml turbidimetrically. This biofilm suspension was diluted in the MH tubes (1:50) for the time-kill assay. Tubes were incubated at 37 °C with shaking and samples were taken at the following time points after the beginning of incubation: 15 min, 30 min, 45 min, 1 h, 2 h, 4 h and 6 h. Each sample was serially diluted and plated. The lower limit of detection was 30 CFU/ml (1.5 × log_10_ CFU/ml). Previous experiments demonstrated that the carryover effect of the peptides in the most concentrated dilution used for viable counts (a 10^−1^ dilution) was negligible.

To quantify the killing efficiency of the treatments, the parameter T_3log_ (defined as the time required for the treatment to decrease 3 logs the initial inoculum) was used.

### Susceptibility assays on biofilms

#### Microplate based assay (static flow regime)

In the microplate based assay, biofilms were grown with no fluid shear and the anti-biofilm activity of peptides was assessed as previously described [55] with some modifications. Briefly, overnight cultures of *P. aeruginosa* PAO1 in TSB were adjusted at 5 × 10^7^ CFU/ml turbidimetrically. This suspension was diluted in TSB (1:100) and 100 μl aliquots were added to the wells of a 96-well plate. After 24 h incubation at 37 °C, planktonic bacteria were removed by gently inverting the plate and then the biofilm-containing wells were washed with saline (0.85 % NaCl). The antimicrobial compound was serially diluted in Mueller Hinton (MH) at final concentrations higher than its planktonic MIC and 200 μl of each dilution was added to the wells. After 24 h incubation at 37 °C, biofilms were stained with either MTT (Sigma, St. Louis, MO, USA) or crystal violet (CV). As control, duplicate biofilms were incubated with MH containing no antimicrobial.

To quantify the disinfection (or bactericidal) activity of the antimicrobials on the biofilms, the MTT assay [56] was used and performed as follows. After the treatment, the biofilms were rinsed with saline and 200 μl of a 0.5 mg/ml dilution of MTT in MH, was added to the wells. Incubation of biofilm with MTT was carried out during 4 h at 37 °C with no shaking. As a measure of cell viability, the conversion of MTT to a tetrazolium salt was determined by solubilizing the salt in 100 μl/well of DMSO and reading the absorbance of the resulting solution at 540 nm.

The ability of the antimicrobials to remove the biofilm attached to the microplate well was determined by CV staining [55, 57]. For this purpose, the treated biofilms were stained with CV during 5 min at room temperature. After the incubation, the excess of stain was rinsed with saline and the CV attached to biofilm was dissolved with ethanol (95 % v/v). The absorbance was measured at 595 nm.

Both the disinfection and removal activity of antimicrobials were expressed as “activity percentage”, a parameter that was calculated using the following formula:

$$ Activity\kern0.26em  Percentage=\frac{\left(C-B\right)-\left(T-B\right)}{\left(C-B\right)}\times 100 $$

Where C is the absorbance value of the control well with non-treated biofilm, T corresponds to the absorbance value of the well with treated biofilm and B is the blank well (i.e. with no biofilm).

#### CDC reactor based assay (dynamic flow regime)

Biofilm growth was also induced under high fluid shear using the CDC-reactor (model CBR 90–1, BioSurface Technologies Corp., Bozeman, MT) as described before [58]. Briefly, 1 ml of an overnight culture of *P. aeruginosa* PAO1 pMF230 was inoculated into 350 ml of TSB and this suspension was used to fill the reactor chamber. After 24 h of growth in batch culture at 37 °C with magnetic agitation, a continuous flow of TSB (3 g/l) was applied to the reactor chamber at a rate of 11 ml/min. Under these conditions, a dense biofilm develops on the surface of small disks called coupons that are constantly bathed in fresh culture medium. After 24 h incubation, the coupons were removed from the chamber and planktonic cells were eliminated by rinsing the coupons with saline. Then, the coupons were immersed in treated with 10 mL of 20 mM phosphate buffer, pH 7, containing different concentrations of the antimicrobial (1, 2 and 10 times their MIC) and incubated at 37 °C for 10 or 60 min. Finally, the coupons were rinsed and processed for colony counting or microscopic visualization as follows.

For the colony counting method, biofilms were detached from the coupon surface by scraping coupons with a sterile wooden stick. Then, biofilm cells were suspended in saline, samples were homogenized at 20500 rpm using Ultraturrax T2 (Janke & Kunkel, IKA labortechnik; Staufen Germanyeterogeneitand plated out for counting. These count values were used to calculate the so called Log Density of the coupon which corresponds to the CFU/cm^2^ of biofilm cells attached to the coupon. In turn, Log Density allowed the determination of Log_10_ reduction (LR), which was defined as the difference of Log Density between the untreated and the treated biofilm. The experiments were performed in duplicate and two coupons were treated with the same peptide concentration in each experiment. The ANOVA Scheffé statistical test was used to assess the efficacy of the treatments.

For the microscopic assessment of anti-biofilm activity, biofilms grown and treated on coupons as detailed above were soaked in a 120 μM solution of propidium iodide (PI, live/dead® Baclight™ bacterial viability kit, Invitrogen, Carlsbad, CA, USA) in filter-sterilized distilled water and incubated for 15 min at room temperature. Then, coupons were rinsed in filter-sterilized distilled water and their surface was examined by fluorescence microscopy using FITC filter (green fluorescence: λ_excitation_, 499 nm; λ_emission,_ 519 nm) and TRITC filter (red fluorescence: λ_excitation_, 552 nm; λ_emission_, 578 nm). To evaluate the ability of the antimicrobial to penetrate into the biofilm structure, biofilms attached to coupons and treated with the most active antimicrobials were incubated with PI as detailed above and then examined with a confocal scanning laser microscope (Leica TCS NT, Solms, Germany). Samples were excited at 480 nm and the emission was measured at 500–550 nm for the green channel, whereas for the red channel the excitation and emission wavelengths were 568 nm and 590–650 nm, respectively. Microscope images were analyzed with the software Imaris® × 64 5.7.2. (Bitplane, Zurich, Switzerland).

### Cytotoxicity assays

Cytotoxicity was assayed on HeLa cells using the MTT [3-(4,5-dimethylthiazoyl-2-yl) 2,5 diphenyltetrazolium bromide protocol [56]. Cells were grown in Dulbeccos’s modified Eagle’s medium (DMEM) and adjusted to a concentration of 10^5^ cells/ml (MTT) in 96 wells polystyrene plates. Plates were incubated for 24 h at 37 °C in 5 % CO_2_, then supplemented with increasing amounts of peptides diluted in 2 mM Hepes pH 7.2, and incubation carried on for 24 h under the same conditions. After this time, filter-sterilized MTT was added in different plates. After 3 h, the content of the wells was gently solved and mixed with 100 μl of DMSO (MTT). Absorbance was measured at 540 nm. Compound cytotoxicity was expressed as LC50, the concentration of the compound that is lethal to 50 % of the cells.

## Abbreviations

MIC: Minimum inhibitory concentration
MBC: Minimum bactericidal concentration
AMP: Antimicrobial peptides
MTT: 3-(4,5-dimethylthiazol-2-yl)-2,5-diphenyltetrazolium bromide
CV: Crystal violet
CLSM: Confocal laser scanning microscopy
CFU: Colony forming unit
TSB: Tryptic soy broth
MH: Mueller Hinton
LB: Luria Bertani
GFP: Green fluorescence protein
CLSI: Clinical Laboratory Standard
NCCLS: National Committee for Clinical Laboratory Standard
LR: Log10 reduction
PI: Propidium iodide
DMEM: Dulbeccos’s modified Eagle’s medium
LC50: Concentration of the compound that is lethal to 50 % of the cells
